# The state of African surgical research capacity: highlighting the current efforts, challenges, and recommendations – Editorial

**DOI:** 10.1097/JS9.0000000000000216

**Published:** 2023-02-16

**Authors:** Andrew A. Wireko, Pearl Ohenewaa Tenkorang, Favour Tope Adebusoye, Aashna Mehta, Jyi Cheng Ng, Owusu Yaa Asieduwaa, Anastasia Fosuah Debrah, Victor Nkemsinachi Oti, Toufik Abdul-Rahman, Vladyslav Sikora

**Affiliations:** a Sumy State University, Sumy; b Kyiv Medical University, Polish Campus, Kyiv, Ukraine; c Faculty of Medicine, University of Debrecen, Debrecen, Hungary; d Faculty of Medicine and Health Sciences, University of Putra, Malaysia; e Faculty of Pharmacy and Pharmaceutical Sciences, Kwame Nkrumah University of Science and Technology, Kumasi; f University of Ghana Medical School, Accra, Ghana

*Dear Editor*,

More than 60% of the world’s population lacks access to safe and affordable surgical care^1^. Surprisingly, safe and low-cost surgery access is unavailable to more than 94% of Africans. Approximately 143 million surgical procedures are needed to save lives in low-income and middle-income countries^1^. Only 6% of the world’s 313 million surgical procedures performed each year are performed in the world’s poorest nations, where the needs are greatest. In 2010, surgical conditions killed nearly 17 million people, outnumbering deaths from malaria, tuberculosis, and HIV/AIDS combined^1^.

Research has revolutionized health care by aiding in the prevention and curing of diseases. Surgical research has focused on the study of surgical techniques, procedures, and technologies. It has improved the safety, effectiveness, and efficiency of surgical interventions and has developed new techniques and technologies for treating various medical conditions^2^. Furthermore, surgical research is an important field of study with the potential to improve patient outcomes, reduce morbidity and mortality, and advance the field of surgery^2^.

Despite the fact that Africa has the most surgical needs, the continent’s research efforts have been woefully inadequate in comparison to the global standard. For more than two decades, African governments and international communities have made tremendous efforts to bridge these surgical research gaps; however, low-income countries (LICs), particularly African nations, account for less than 15% of all surgical papers published globally^1^. Sooryamoorthy *et al*.^3^ recently published a study highlighting how Africa contributes 7.6% to the world of science and, importantly, one-third of all international publications in tropical medicine (Fig. 1).

**Figure 1:**
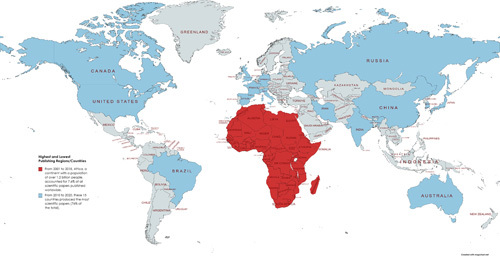
Countries with the highest number of scientific publications in comparison to African nations. The African continent has nearly 1.2 billion people accounting for just 7.6% of all scientific papers published worldwide between 2001 and 2018. The top 15 countries produced 76% of the world’s papers (from 2010 to 2020), highlighting huge research gaps and inequities globally (created with Mapchart.net)^3^,^5^.

A recent study of fracture surgery papers in global publications found that only 0.1% of the papers were attributed to LICs over a decade, whereas high-income countries had 86.6% of the publications^4^. One-third of the publications were written independently by LIC authors, while the remaining two-thirds were co-authored by high-income country and middle-income country authors^4^. It has been extremely difficult to obtain quality data and published research papers to critically assess the surgical needs of the African population.

Significant efforts have been made in recent years to close these research gaps. Several organizations, including the WHO, the African Surgical Outcomes Study group, the World Federation of Neurosurgical Societies, the African College of Surgeons of East, Central, and Southern Africa, and many others, have emerged and improved collaborative initiatives to aid surgical research in Africa^6^–^8^. These fantastic initiatives have provided rigorous epidemiological data on surgical activity, perioperative outcomes, and surgical workforce density in Africa^6^. Furthermore, a number of student surgical institutions have emerged, with significant efforts made to raise awareness of surgery research and improve surgical outcomes. An example is the establishment of the Aspect Research Incubator Program by the Future African Neurosurgeons to help aspiring academic neurosurgeons improve their research capacities^8^. Aspect Research Incubator Program has had a lot of success so far, with over 28 published papers and 20 conference abstract presentations^8^.

Unfortunately, the challenges that African surgical research capacity faces far outweigh the efforts and advances. Surgical research in Africa is hampered primarily by a lack of human resources, infrastructure, and funding (Fig. 2)^4^,^6^,^8^. When it comes to establishing a successful and long-term research activity, the value of human resources cannot be overstated. Unfortunately, many African countries face significant human resource shortages, particularly in the health sector. The few that remain are sometimes under-skilled, uncommitted, and exhibit poor team commitments, as well as poor research leadership to get things done properly^6^.

**Figure 2:**
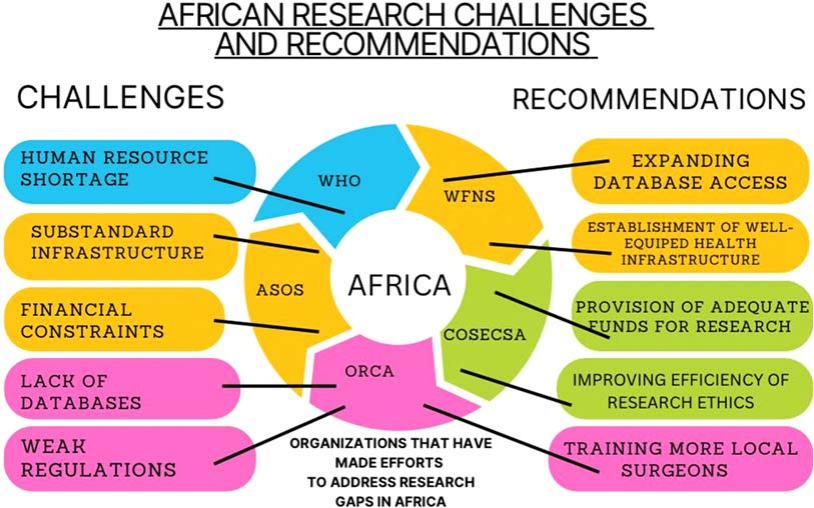
The efforts of various organizations, as well as the challenges that Africa still faces in surgical research and recommendations. ASOS, African Surgical Outcome Study; COSECSA, College of Surgeons of East, Central and South Africa; ORCA, Orthopedic Research Collaboration in Africa; WFNS, World Federation of Neurosurgical Societies (created with Canva).

Aside from the aforementioned challenges, insufficient poor health standards, substandard health infrastructure, poor internet, and a lack of well-equipped hospitals and clinical laboratories all contribute to high-quality research limitations (Fig. 2)^4^,^6^,^8^. As a result, it is difficult to conduct high-quality, large-scale surgical research. To make matters worse, Kanmounye *et al*.^8^ highlighted a lack of funding for surgical research involving human trials, even to the point of being unable to pay for article processing charges when researchers intend to publish their findings due to financial constraints. Furthermore, the process of publishing surgical research findings can be difficult at times because surgeons struggle to get their work approved for publication due to the poor quality of research conducted on the continent. Payments for conferences, as well as international travel for presentations, have all proven to be a significant challenge^8^. All of the challenges have had a significant impact on the academic productivity of surgeons in Africa. Furthermore, most African medical students and surgeons are unable to access the majority of full-text papers through their institutions when they want to read and update their knowledge on new findings in literature or possibly conduct secondary research. This is probably due to the fact that individuals and institutions cannot afford the fees for access to these massive journal publications. As a result of all of these research constraints, students and aspiring surgeons are disinclined to contribute to the surgical world of academia.

Another issue in Africa is the lack of regional and national databases on surgical outcomes (Fig. 2). Most hospitals have extremely poor record keeping, resulting in a lack of or insufficient data, making it difficult to assess the effectiveness of various surgical interventions and identify areas for improvement^6^. The scarcity of data makes developing evidence-based surgical practice guidelines in Africa difficult. Also poor regulatory requirements and ethical concerns also make conducting research in low-resource settings difficult. Obtaining regional and national approval to conduct research in many African countries has been difficult^6^. To make matters worse, surgeons’ schedules, particularly in Africa, can be quite hectic, making it difficult for them to devote time to research. Africa has very few surgeons managing a large population, making it difficult for them to perform other duties aside from patient care. Furthermore, due to the lack of an institutionalized research framework, surgeons may find it difficult to find colleagues for surgical research initiatives, either at their own institutions or at other universities.

In order to improve the African surgical research standards, African governments should prioritize high-quality, safe, and equitable surgical care, with research as a key area for advancement. These governments and funders should prioritize the development of national research capacity by providing adequate funding and resources to support the work of health researchers, such as competitive salaries, benefits, and opportunities for career development for young researchers. Putting these resources in place will also help to address the issue of health worker migration. In order to maintain effective healthcar e quality standards, new and well-equipped health infrastructure should be built and expanded (Fig. 2). Furthermore, well-planned strategies must be implemented to ensure equitable access to all available resources.

To improve the long-term quality of surgery and healthcare delivery in Africa, African governments and the international community should prioritize fostering the development of local and rural surgical research capacity. This can be accomplished by training more local surgeons and other health workforce, as well as providing massive support for the establishment of research infrastructure in these neglected populations (Fig. 2). Training more locals will increase research personnel and improve the overall quality of research and patient care on a long-term basis. Improved incentives and research opportunities for surgeons are needed to change the unreceptive culture of African surgeons toward research and collaboration, as well as to improve the framework and network for surgical research. There should be more promotion of knowledge and resource sharing among researchers and institutions, both within and between African nations and other countries. This could help to ensure that research findings are widely disseminated and that the benefits of research are distributed more evenly around the world. Furthermore, encouraging collaboration and networking among African researchers can help to increase the impact of surgical research in the region. This could include establishing long-term networks of Africans from all African nations to effectively collaborate.

## Ethical approval and consent to participate

Not applicable.

## Sources of funding

None.

## Author contribution

A.A.W.: conceptualization ideas. All authors were involved in this process of data curation, writing of the initial draft, review and editing, and final draft.

## Conflicts of interest disclosure

The authors declare no conflicts of interest.

## Research registration unique identifying number (UIN)

1. Name of the registry: NA.

2. Unique identifying number or registration ID: NA.

3. Hyperlink to your specific registration (must be publicly accessible and will be checked): NA.

## Guarantor

A.A. Wireko.

## Consent for publication

Not applicable.

## Data availability

No new data were generated.
